# Membrane-initiated actions of estradiol (E2) in the regulation of LH secretion in ovariectomized (OVX) ewes

**DOI:** 10.1186/1477-7827-8-40

**Published:** 2010-05-10

**Authors:** J Alejandro Arreguin-Arevalo, Ryan L Ashley, Elizabeth R Wagenmaker, Amy E Oakley, Fred J Karsch, Terry M Nett

**Affiliations:** 1 Department of Biomedical Science, Colorado State University, Fort Collins, Colorado 80523, USA; 2 Instituto Nacional de Investigaciones Forestales Agrícolas y Pecuarias, Centro de Investigación Regional Golfo Centro, Veracruz, México; 3 Department of Molecular and Integrative Physiology, University of Michigan, Ann Arbor, Michigan 48109, USA; 4 Department of Physiology and Biophysics, University of Washington, Seattle, WA 98195, USA

## Abstract

**Background:**

We demonstrated that E2 conjugated to BSA (E2BSA) induces a rapid membrane-initiated inhibition of LH secretion followed hours later by a slight increase in LH secretion. Whether these actions of E2BSA are restricted to the pituitary gland and whether the membrane-initiated pathway of E2BSA contributes to the up-regulation of the number of GnRH receptors during the positive feedback effect of E2 were evaluated here. We have shown that the suppression of LH secretion induced by E2 and E2BSA is the result of a decreased responsiveness of the pituitary gland to GnRH. In this study we further tested the ability of E2BSA to decrease the responsiveness of the pituitary gland to GnRH under the paradigm of the preovulatory surge of LH induced by E2.

**Methods:**

For the first experiment GnRH and LH secretions were determined in samples of pituitary portal and jugular blood, respectively, in ewes treated with 12 mg E2BSA. In the second experiment, the number of GnRH receptors was quantified in ewes 12 h after administration of 25 micrograms E2 (the expected time for the increase in the number of GnRH receptors and the positive feedback effect of E2 in LH secretion) or 12 mg E2BSA. In the third experiment, the preovulatory-like surge of LH was characterized in ewes injected with 25 micrograms E2 alone or followed 8 h later (before the beginning of the LH surge) with 60 mg E2BSA.

**Results:**

a) the decrease in LH secretion induced by E2BSA was not accompanied by changes in the pulsatile pattern of GnRH, b) E2BSA increased the number of GnRH receptors, and c) the presence of E2BSA in E2-treated ewes delayed the onset, reduced the length, and decreased the amount of LH released during the preovulatory surge of LH.

**Conclusions:**

a) the rapid suppression of LH secretion induced by E2BSA is mediated only via a direct action on the pituitary gland, b) E2 acting via a membrane-initiated pathway contributes to increase the number of GnRH receptors and, c) administration of E_2_BSA near the beginning of the pre-ovulatory surge of LH delays and reduces the magnitude of the surge.

## Background

The ability of E_2 _to induce a preovulatory surge of LH [1-5] is the result of actions of E_2 _on both the pituitary gland and the hypothalamus. In the pituitary gland E_2 _increases the sensitivity to GnRH by inducing synthesis of GnRH receptors on gonadotropes [6,7]. In the hypothalamus E_2 _induces a sustained surge of GnRH [5,8]; possibly by activating E_2_-receptive neurons that in turn activate GnRH neurons [9,10]. Two decades ago, evidence for a short-term negative feedback of E_2 _on secretion of LH was reported [2,11]. From studies in a variety of endocrine paradigms it has been shown that both the pituitary gland [2,12-14] and the hypothalamus mediate the rapid inhibition of LH by E_2 _[5,15-18] and that the transition from negative to positive feedback in the pituitary gland is an important component for onset of the preovulatory surge of LH [19].

The ability of E_2 _to modulate cellular function via novel membrane-initiated (non-genomic) mechanisms [20-23] opens new avenues to study a potential interaction between positive and negative feedback effects of E_2 _on secretion of LH. E_2 _conjugated to BSA has been used widely to mimic non-genomic biological responses of E_2 _in a variety of tissues [24-29]. The rapid biological responses induced by E_2_BSA are interpreted to be as an action initiated at the cell membrane, acting through specific binding sites that are linked to intracellular signal transduction proteins.

We previously demonstrated that either free E_2 _or E_2_BSA induced a rapid decrease in secretion of LH in OVX ewes [30] and in primary cultures of pituitary cells [13], suggesting a direct action on the pituitary. In the current study, we conducted experiments to examine if membrane-initiated actions of E_2_-BSA in regulating LH secretion were restricted to the level of the pituitary gland. In the first experiment we tested the hypothesis that E_2_BSA would rapidly inhibit pulsatile secretion of LH without a corresponding decrease in GnRH release from the hypothalamus.

Genomic and non-genomic actions of E_2 _frequently converge to induce the same cellular response [20-23]. Under this comprehensive model, E_2 _binds to both nuclear receptors and to putative plasma membrane binding sites. Binding of E_2 _to nuclear receptors leads to an interaction with estrogen response elements (ERE) in target genes resulting in regulation of gene transcription. In contrast, binding of E_2 _to membrane binding sites activates specific signal transduction pathways resulting in a more efficient up- or down-regulation of gene transcription. We previously reported that conjugated forms of E_2 _did not induce a preovulatory-like surge of LH; instead a slight but significant increase in serum concentrations of LH was detected at the expected time of the massive release of LH [30]. Since it is unlikely that a molecule as large as E_2_BSA crosses the blood-brain barrier to induce a preovulatory-like surge of GnRH, we speculated that the slight increase in secretion of LH is the result of a direct action on the pituitary gland to increase the number of GnRH receptors [30]. In the second experiment of this study, we examined the hypothesis that E_2_BSA will increase the number of GnRH receptors in OVX ewes.

The ability of conjugated E_2 _to diminish the responsiveness of the pituitary gland to GnRH has been demonstrated in primary cultures of ovine pituitary cells [13]. The third experiment described herein further evaluates the ability of E_2_BSA to override the massive release of LH induced by a single bolus of free E_2_. The hypothesis is that E_2_BSA by acting at the plasma membrane level could diminish the responsiveness of the pituitary gland during the surge of GnRH induced by E_2_.

## Methods

### Preparation of media and stock solution

Free E_2 _was removed from E_2_BSA (Steraloids Inc., Newport, RI) by ten consecutive extractions with diethyl ether [31]. As previously shown [13], less than 1% of the weight of the conjugate was removed as free E_2_, and the amount of free E_2 _remaining in the E_2_BSA conjugate was substantially less than the concentration needed to elicit a physiological response. D-Ala^6^-desGlyNH_2_^10^-GnRH-ethylamide (D-Ala^6^-GnRH-EA) and E_2 _were purchased from Sigma (Sigma-Aldrich, Inc., Saint Louis, MO). E_2 _was freshly dissolved in ethanol the day of treatment and emulsified in mineral oil, whereas E_2_BSA was dissolved in sterile saline solution.

### Stability of E_2_BSA in serum

A caveat of using E_2_BSA as a tool to study non-genomic actions is the potential instability of the conjugate. Our assumption was that if the conjugate breaks down, the resulting free (non-conjugated) E_2 _should be detectable in serum after organic solvent extraction. Therefore, to evaluate the ability of E_2_BSA to remain conjugated in serum, OVX ewes (n = 3) received 174 nmoles E_2_BSA (based on the molecular weight of BSA) or 92 nmoles of E_2 _intramuscularly. This dose of E_2_BSA induces a rapid suppression of LH secretion; followed by a preovulatory-like surge of LH [30]. Blood samples were collected at 0, 1, 2, 6, 10, 16, and 24 h after treatment. Non-conjugated E_2 _was removed from serum samples by two consecutive extractions with diethyl ether and immunoreactive E_2 _was quantified by RIA [31]. The intra-assay coefficient of variation was 12% and the assay sensitivity was 0.18 pg/per tube.

### Measurement of GnRH receptors

GnRH receptors were quantified as previously described [32]. The pituitary gland was divided mid-sagitally and half of the anterior pituitary was homogenized (100 mg/ml) in Tris buffer (10 mM Tris, 1 mM CaCl_2_, and 0.1% [w/v] bovine serum albumin [pH = 7.4]). The homogenate was centrifuged at 30,000 × g for 30 min at 4 C and supernatant was decanted. The top two layers of the pellet were resuspended in 5 ml of Tris buffer and rehomogenized in a Dounce homogenizer. The tissue was centrifuged at 30,000 × g for 30 min at 4 C and the top layer of the pellet was removed and resuspended in 1 ml of Tris buffer. Protein concentration was determined using the BCA protein assay kit (Pierce, Rockford, IL). [^125^I]-D-Ala^6^-GnRH-EA was prepared by the glucose oxidase-lactoperoxidase technique [33]. Fifty and 100 μl of crude membrane preparations were incubated with 0.43 nM [^125^I]-D-Ala^6^-GnRH-EA in a total volume of 150 μl for 4 h at 4 C. At the end of the incubation, the membranes were diluted with 3 ml of ice-cold Tris buffer and centrifuged for 10 min at 30,000 × g. The supernatants were decanted and the radioactivity remaining in the pellet was quantified. Number of GnRH receptors were quantified using a standard curve technique as described previously [32]. All samples were quantified in one assay and the inter-assay coefficient of variation was 2.8%.

### Animals and experimental protocol

Procedures involving animals in experiment 1 were approved by the Committee for the Use and Care of Animals at the University of Michigan whereas procedures in experiments 2 and 3 were approved by the Colorado State University Animal Care and Use Committee and complied with National Institutes of Health (NIH) guidelines. All experiments were carried out between October and January (breeding season) and ewes were ovariectomized (OVX) at least 2 months before treatment. In a preliminary experiment a single im bolus of E_2_BSA induced a decrease in secretion of LH followed by a slight rise in serum concentrations of LH. Therefore steroids were administered intramuscularly.

### Experiment 1

Mature Suffolk ewes (n = 6) were fitted with an apparatus for collection of pituitary portal blood, according to procedures described previously [34]. After a 2-wk recovery period, ewes were equipped with two indwelling jugular catheters, one for collecting peripheral blood and one for infusing heparin saline (250 U/min). Samples of pituitary portal and jugular blood were collected through a remote automated sampling system that allows continuous collection of samples [34]. Pituitary portal and jugular blood samples were withdrawn continuously and separated into 10-min fractions for analysis of GnRH [35] and LH [36] by radioimmunoassay. GnRH quantification was carried out at University of Michigan whereas LH was quantified at Colorado State University. Pituitary portal blood was dispensed into tubes containing ice-cold bacitracin, extracted with methanol (~300 μl plasma) within 1.5 h of sampling and stored at -80 C until analysis of GnRH. After sample collection, ewes were euthanized and the pituitary was inspected for appropriate placement of the lesion in the portal vasculature, to corroborate adequate collection of pituitary portal blood. One ewe (data not shown) was excluded since there was not an obvious lesion on the pituitary gland and GnRH values were exceedingly low and not clearly episodic. For GnRH the intra-assay coefficient of variation was 4.3% and the assay sensitivity was 0.09 pg/tube (3 assays). For LH the intra-assay coefficient of variation was 5% and the assay sensitivity was 30 pg/tube. To evaluate the ability of E_2_BSA to suppress the pulsatile pattern of GnRH, six ewes received 12 mg E_2_BSA intramuscularly. Pituitary portal and peripheral blood were separated into 10 min fractions from 4 h before to 4 h after treatment with E_2_BSA. GnRH and LH pulses were defined as an increase in hormone concentration higher than the mean concentration detected during the 4 h pre-treatment period within an individual followed by at least one descending hormone concentration. GnRH and LH pulse amplitudes were defined as the difference between the peak and its preceding nadir. Because the sampling procedure measures GnRH only in its initial pass through the portal system (GnRH re-circulating from periphery is undetectable) and because many GnRH pulses were split between two consecutive 10-min samples [GnRH pulses last 5-6 min [37]], the amplitude of a given GnRH pulse was calculated by adding all values greater than baseline and subtracting the value of the preceding nadir. GnRH in pituitary portal blood was calculated as a collection rate (pg per minute) rather than concentration. This minimizes error due to changes in rate of portal blood withdrawal caused by changes in head position or contamination of portal blood with CSF (cerebrospinal fluid) or peripheral blood. The latter was judged to be minimal based on little or no blood aspirated into the sampling lines during the hour prior to lesion of the portal vessels; CSF contamination was negligible based on hourly hematocrit measures during sampling.

### Experiment 2

Mature Western-range ewes were fitted with a jugular cannula to withdraw blood samples. To determine if an increase in the number of GnRH receptors was associated with the increase in LH induced by E_2_BSA, ewes (n = 6) received 92 nmoles E_2 _(in 3 ml of safflower oil) or 174 nmoles E_2_BSA (in 3 ml saline solution). Sham injections of saline solution or safflower oil were administrated to E_2_- and E_2_BSA-treated ewes, respectively. This dose of E_2_BSA induces a slight but significant increase in serum concentrations of LH at the expected time of the massive release of LH [30,38]. Blood samples were collected before administration of estrogenic compounds and every 2 h from 6 to 12 h after treatment. To minimize the effect of GnRH released during a preovulatory-like surge of LH on the number of GnRH receptors, pituitary glands were collected at the beginning of the time of the expected surge of LH (12 h after treatment). This interval proved to be sufficient to induce the highest number of GnRH receptors in primary cultures of ovine pituitary cells incubated with E_2 _or E_2_BSA [39]. Concentrations of LH were determined in one assay. The intra-assay coefficient of variation was 5% and the assay sensitivity was 36 pg/tube.

### Experiment 3

The objective was to test the hypothesis that E_2_BSA is able to disrupt the preovulatory-like surge of LH induced by E_2_. Disruption of the preovulatory-like surge of LH would open a new avenue to study conjugated forms of E_2 _as a potential method of contraception that might be more specific than the current steroidal contraceptives. We have demonstrated that conjugated E_2 _is able to reduce GnRH-induced release of LH in primary cultures of ovine pituitary cells [13] and to suppress pulsatile secretion of LH in OVX ewes for at least 10 h under basal secretion of GnRH [30]. We speculated that E_2_BSA, by acting at the plasma membrane level, could diminish the responsiveness of the pituitary gland to the surge of GnRH induced by E_2_. We presumed that, in order to overcome the stimulatory effect of the massive release of GnRH, a higher dose of E_2_BSA would be necessary. Thus, we administered E_2_BSA to a dose ~10 times higher than E_2 _(based on molecular weight of BSA). Due to the variability in the magnitude of LH surge induced by E_2_, in a preliminary study the magnitude of the LH surge was determined in mature Western-range ewes. Animals were ranked based on the mean LH during the surge and then assigned to either a high or a low E_2_-responsive group (n = 6 per group). At least one month later, ewes (n = 12) ranked within each E_2_-responsive group were alternately assigned to received an im injection of 92 nmoles E_2 _at time zero. Six of these ewes received an im injection of 870 nmoles E_2_BSA (in 3 ml saline solution) or saline solution 8 h after injection of E_2_. Blood samples were collected every hour from 4 h before to 28 h after treatment and LH was quantified. Intra- and inter-assay (10 assays) coefficients of variation for LH were 5 and 10%, respectively. The assay sensitivity was 32 pg/tube. The effect of E_2 _alone or together with E_2_BSA on the preovulatory-like surge of LH was evaluated using five parameters: 1) Mean LH, 2) Amplitude; defined as the highest concentration of LH, 3) Area under the curve during the surge of LH (AUC), 4) Length of the preovulatory-like surge, defined as the period between the onset and the end of the LH surge, and 5) Interval from administration of E_2 _to the onset of the massive release of LH. A preovulatory-like surge of LH was described as a concentration of LH equal to or higher than the pre-treatment mean LH plus 2 standard deviations detected within an individual (surge onset), followed by increasing concentrations of LH reaching an amplitude of at least 40 ng/ml, and remaining over onset concentration for at least 6 h. The end of a surge of LH was described as a descending concentration of LH, equal to or lower than the pre-treatment mean LH plus 2 standard deviations detected within an individual.

A preovulatory-like surge of LH was defined as the period during which an increasing concentration of LH equal to or higher than the pre-treatment mean LH plus 2 standard deviations first is detected within an individual, reaching amplitude of at least 40 ng/ml, and remaining over onset concentration for at least 6 h. The end of a surge of LH was defined as a descending concentration of LH that no longer met the criteria for a surge.

### Data analysis

Data were subjected to analysis of variance (ANOVA) using the general linear model of SAS [40]. Serum concentrations of LH (experiments 1 and 2) and E_2_, as well as GnRH collection rate (experiment 1) were evaluated by repeated measures analysis. Number of pulses of GnRH and LH were subjected to arcsine transformation. Changes in the number of GnRH receptors were analyzed in a completely randomized design. Variables related to the preovulatory-like surge of LH (experiment 3) were analyzed in a completely randomized design with a factorial arrangement. The factors included in the analysis were treatment, level of responsiveness to E_2 _(high and low) and their interaction. When differences among treatment means were detected, they were separated using the Least Significant Difference (LSD) procedure. If variances were not homogeneous, data were Log_10 _transformed.

## Results

To demonstrate the stability of E_2_BSA in serum, ewes received an im injection of either E_2 _(92 nmoles) or E_2_BSA (174 nmoles) and free, unconjugated E_2 _was quantified. In E_2_-treated ewes serum concentrations of E_2 _reached a peak (P < 0.01; 206 ± 15 pg/ml) 1 h after administration, remained above baseline for at least 16 h and then returned to pre-treatment concentration (1.0 ± 0.3 pg/ml). Conversely, in E_2_BSA-treated ewes there was no change in serum concentrations of immunoreactive E_2 _and it remained similar to pre-treatment levels during the period evaluated (0.7 ± 0.3 vs. 1.0 ± 0.3 pg/ml).

### Experiment 1

**Figure 1:**
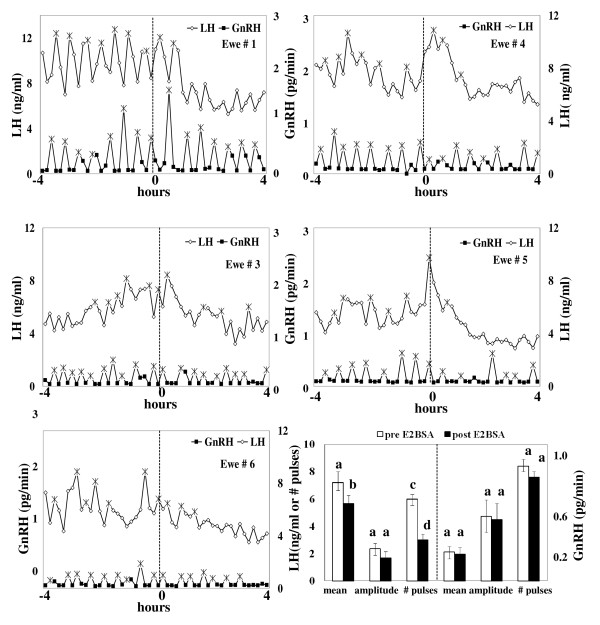
**Effect of E_2_BSA on the secretory patterns of LH and GnRH**. (a) Secretory patterns of LH and GnRH in individual ewes during the acute inhibition of LH induced by E_2_BSA. Patterns of LH (open diamonds) and GnRH (closed squares) in individual ewes treated with an im bolus of 12 mg E_2_BSA (hour 0). GnRH and LH were measured in samples of pituitary portal and jugular blood, respectively, withdrawn continuously and separated into 10-min fractions, from 4 h before to 4 h after treatment. (*) is indicative of a pulse. (b) E_2_BSA decreased secretion of LH without affecting the secretory patterns of GnRH during the acute inhibition of LH secretion. Mean plasma rate of GnRH, number of pulses of GnRH and amplitude of pulses (right panel) were measured in pituitary portal blood whereas mean LH, number of pulses and amplitude of pulses of LH (left panel) were measured in jugular blood. Samples were collected at 10-min intervals, from 4 h before (open bars) to 4 h after treatment (solid bars). Data are presented as the mean ± SEM. Comparisons were made within periods. a, b differ (P < 0.02); c, d differ (P < 0.01).

GnRH and LH profiles of individual ewes treated with E_2_BSA are depicted in Figure 1a. Prior to treatment, GnRH and LH pulses were readily detected in all ewes. In 4 of 5 ewes, LH pulses were less evident after administration of E_2_BSA. In the remaining ewe (# 3), LH decreased steadily during the post-E_2_BSA period. Compared to pre-treatment period (Figure 1b), E_2_BSA decreased mean plasma concentrations of LH (P < 0.02) and the number of pulses of LH (P < 0.01); however, no changes were detected in the amplitude of pulses of LH. In contrast to LH, E_2_BSA did not alter the mean collection rate of GnRH, the number of pulses of GnRH, or the amplitude of pulses of GnRH (Figures 1a and 1b).

### Experiment 2

**Figure 2:**
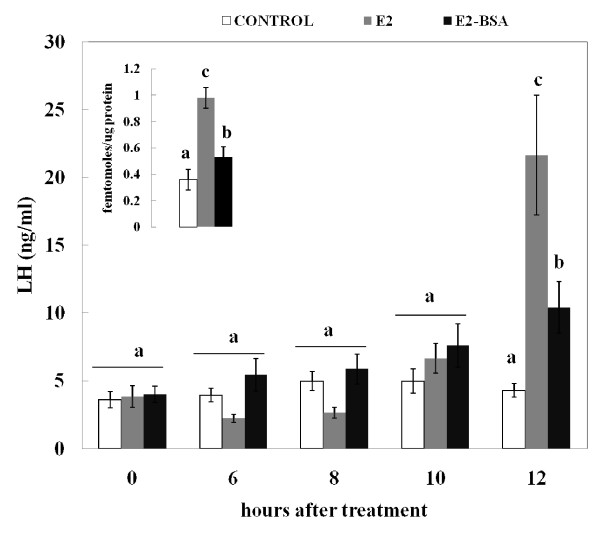
**E_2 _and E_2_BSA increased the number of GnRH receptors and LH secretion at the expected time for the beginning of the preovulatory-like surge of LH**. Effect of Ewes (n = 6) received an im injection of saline solution (control), E_2_, or E_2_BSA at time zero. Mean serum LH was measured at 0, 6, 8, 10, and 12 h after treatments. Comparisons were made within and among treatments. a, b differ (P < 0.05), c differs from a/b (P < 0.01). Insert depicts changes in the number of GnRH receptors in ovine pituitary glands from ewes 12 h after administration of treatments. Bars with unlike subscripts differ (P < 0.05). Data are presented as the mean ± SEM.

Twelve hours after treatment with E_2 _or E_2_BSA the number of GnRH receptors increased (P < 0.05) compared to controls (Figure 2 insert). The increase was higher (P < 0.05) in E_2_-treated ewes compared to ewes treated with E_2_BSA (Figure 2 insert). Before treatments, serum concentrations of LH were similar among groups (Figure 2). Twelve hours after administration of the estrogenic compounds, serum concentrations of LH were elevated (P < 0.05) in E_2_- and E_2_BSA-treated ewes and were higher (P < 0.01) in ewes treated with E_2 _than in ewes treated with E_2_BSA (Figure 2).

### Experiment 3

**Figure 3:**
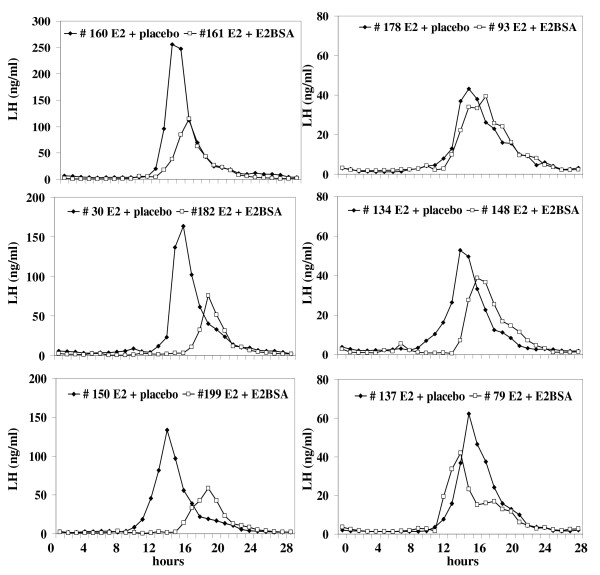
**Secretory pattern of LH in individual ewes showing the effect of a high dose of E_2_BSA on the preovulatory-like surge of LH induced by E_2_**. In a preliminary study the magnitude of the LH surge in response to E_2 _was determined; ewes were ranked based in the mean LH during the surge induced by E_2_, then assigned to either a high (LH profiles in the left panel) or a low (LH profiles in the right panel) E_2_-responsive group (n = 6 per group). Approximately one month later, ewes (n = 12) were given an im injection of 25 μg E_2 _and then assigned alternatively within each responsive group, to receive an im injection of 60 mg E_2_BSA (open squares) 8 h after injection of E_2 _or saline solution (placebo; closed diamonds). The negative impact to E_2_BSA on the massive release of LH induced by E_2 _was more pronounced in ewes classified as highly responsive to E_2_.

**Figure 4:**
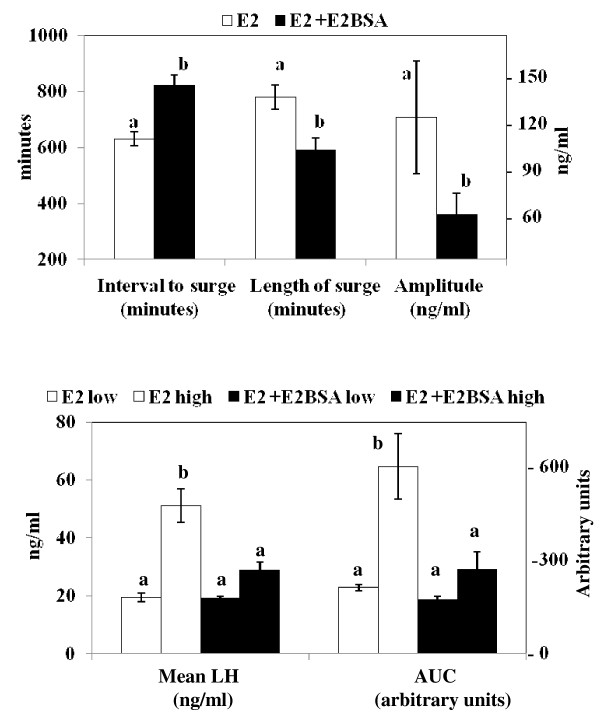
**Effect of E_2_BSA on the preovulatory-like surge of LH induced by E_2_**. Ewes received an im injection of 60 mg E_2_BSA 8 h after injection of E_2 _or saline solution (placebo). Data are presented as the mean ± SEM. Unlike subscripts differ (P < 0.05). (a) E_2_BSA delayed the interval to the surge, reduced the length of the surge, and decreased the amplitude of the surge induced by E_2_, regardless the magnitude of the response to E_2_. (b) E_2_BSA decreased the mean LH and the AUC (area under the curve of LH) induced by E_2_, but only in ewes classified as highly responsive to E_2_.

Figure 3 depicts individual LH profiles during the preovulatory-like surge of LH in OVX ewes treated with E_2 _alone and E_2 _followed 8 h later by E_2_BSA or saline solution. The panels on the left depict the LH profiles of ewes previously classified as highly responsive to E_2_. The panels on the right depict the LH profiles of ewes previously classified as less responsive to E_2_. All ewes treated with E_2 _expressed a surge of LH, starting approximately 12.1 h (725 ± 34 min) after injection of E_2 _with an average length of 11.4 h (685 ± 49 min; Figures 3 and 4a). Intramuscular injection of 60 mg E_2_BSA in ewes pre-treated with E_2 _delayed (P < 0.05; Figure 4a) the onset of the preovulatory-like surge of LH and decreased (P < 0.05; Figure 4a) the length and amplitude of the surge of LH regardless the level of responsiveness to E_2_; E_2_BSA decreased mean serum concentrations of LH and the AUC (i.e. the total amount of LH released) only in ewes classified as highly responsive to E_2 _(P < 0.05; Figure 4b).

## Discussion

We demonstrated previously that equimolar concentrations of free E_2 _or E_2 _conjugated to BSA suppressed the GnRH-induced release of LH in cultures of ovine pituitary cells [13] suggesting a direct action on the pituitary gland. In OVX ewes, a similar decrease in LH secretion was recapitulated by administration of E_2 _or E_2_BSA at equimolar concentrations [30]. In this study we tested the possibility that the decrease in LH secretion induced by E_2_BSA could (at least partially) be mediated by an action at the level of the hypothalamus. We found that the decrease in LH pulse frequency and mean LH induced by E_2_BSA was not accompanied by changes in either pulse frequency or pulse amplitude of GnRH. Therefore, the actions of E_2_BSA on LH secretion seem to occur only at the level of the pituitary gland, presumably by decreasing responsiveness to GnRH. Together with our previous reports [13,30], these data suggest that pharmacological targeting of the pituitary gland with E_2_BSA is sufficient to mimic the rapid inhibitory action of E_2 _on LH secretion. It is worth mentioning that we did not detect a decrease in the pulse amplitude of LH. It is possible that our criterion to detect pulses did not identify small increases in LH concentrations as pulses, or that our sampling was too infrequent to detect a marginal decrease in pulse amplitude. The exclusion of these "low amplitude pulses" may have masked an effect on the amplitude of LH pulses. Nevertheless, since E_2_BSA did not decrease either pulse frequency or pulse amplitude of GnRH, the effect of E_2_BSA on LH secretion is attributed to a direct action on the pituitary gland. The apparent lack of effect of E_2_BSA on the secretory pattern of GnRH provides evidence that E_2_BSA does not have a hypothalamic effect, possibly because it does not cross the blood-brain barrier. In a recent study [41] evidence was provided for the mechanism underlying the rapid inhibitory effect of E_2 _on the GnRH-induced release of LH secretion in primary cultures of ovine pituitary cells. In that study, the increase in cytoplasmic intracellular free calcium concentration ([Ca^++^]i) mediating the GnRH-induced release of LH secretion was abolished by 2 min pretreatment with E_2 _or E_2_BSA, which agrees with the time frame for the beginning of the rapid decrease of LH secretion induced by estrogens. In hypothalamus-pituitary-disconnected ewes, E_2 _increased the number of gonadotropes expressing phosphorylated extracellular signal-regulated-kinases 1 and 2 (pERK-1/2), c-AMP-responsive element-binding protein (pCREB), and serine 473 kinase (pAkt) within 15 to 90 minutes after administration of E_2 _[42]. Whether these and/or other upstream signaling molecules contribute to maintain a decreased responsiveness of the pituitary gland to GnRH (for at least 10 h in our previous study [30]) after administration of estrogens is yet to be evaluated.

A caveat of using E_2_BSA as a tool to study non-genomic actions is the stability of the conjugate, particularly if the conjugate is used in studies in vivo. We demonstrated [30] that conjugated forms of E_2 _mimicked the acute non-genomic action of E_2 _(i.e. rapid decrease of LH secretion), but not the more prolonged, presumably genomic actions (i.e. preovulatory-like surge of LH and decrease in FSH secretion). These data strongly support the conclusion that there is insufficient free E_2 _(i.e. enough to mimic the genomic actions) in the conjugate to have a biological effect. In the current study we provided further evidence on the stability of E_2_BSA in blood. It is known that E_2 _can rapidly regulate secretion of GnRH [8,16,18,43,44]. Consequently, the fact that E_2_BSA did not change the secretory pattern of GnRH strengthens our previous conclusion of the absence of sufficient free E_2 _to induce a physiological response. Furthermore, administration of E_2_BSA to OVX ewes did not raise serum concentrations of free E_2 _over pre-treatment levels during the 24 hours evaluated. Therefore, the actions of E_2_BSA can only be attributed to the conjugated form of E_2_.

In a previous report [30], we showed that infusion of E_2_BSA to OVX ewes did not induce a typical preovulatory-like surge of LH; instead a slight but significant increase in serum concentrations of LH was detected at the time of the expected massive release of LH. The possibility that the slight increase in LH secretion induced by E_2_BSA was the result of an increase in the number of GnRH receptors was evaluated in this study. Indeed, administration of E_2_BSA to OVX ewes increased the number of GnRH receptors in the pituitary gland and it was associated with a slight rise in serum concentrations of LH at the expected time for the beginning of the preovulatory-like surge of LH (12 h after administration of conjugated E_2_). This report provides the first in vivo evidence in ewes of a membrane-initiated pathway as a component of the mechanisms underlying the synthesis of GnRH receptors induced by E_2_. It seems that the non-genomic actions of E_2 _in the pituitary gland are not restricted to the rapid suppression of pulses of LH reported elsewhere [13]. The ability of E_2_BSA to induce both a suppression in responsiveness of the pituitary gland to GnRH and an increase in the number of GnRH receptors is compatible since E_2 _up-regulates the number of GnRH receptors subsequent to the short-term negative feedback [2,6,45,46] and the sum of these two events may be part of the strategy to ensure a buildup of pituitary content of LH for a robust preovulatory surge. Since an ERE is not involved in mediating the actions of E_2_BSA [24,27,47,48] and a canonical ERE has not been reported in the GnRH receptor gene [47-50], we speculate that a membrane-initiated pathway may activate down-stream events facilitating the transcription of GnRH receptors, as well as inhibiting GnRH-induced secretion of LH. As mentioned above, an increase in phosphorylation level of a number of proteins in the gonadotropes has been reported 15 to 90 minutes after administration of E_2 _[42]. The ability of E_2 _or E_2_BSA to increase the level of protein phosphorylation for longer periods would be compatible with our speculation on a membrane-initiated pathway as a component in the synthesis of GnRH receptors number, as well as maintaining the inhibition of LH secretion after the initial decrease. The ability of E_2 _and E_2_BSA to activate upstream signaling molecules for longer intervals has yet to be evaluated.

The ability of E_2_BSA to inhibit responsiveness of primary cultures of ovine pituitary cells to GnRH [13] was the basis for examining its ability to abolish the E_2_-induced massive release of LH in ewes. Based on our previous in vivo study [30] E_2_BSA would decrease responsiveness of the pituitary gland to GnRH within the first hour after E_2_BSA, and it remained decreased for at least 10 h under basal secretion of GnRH. In the current experiment we overlap the period of a decreased responsiveness of the pituitary gland to GnRH to the expected time for the surge of LH by administering E_2_BSA 8 h after E_2 _(2-3 h before the beginning of the preovulatory-like surge of LH) at a dose 10 times higher than previously reported [30]. Under these experimental conditions, E_2_BSA failed to obliterate completely the E_2_-induced massive release of LH. Nevertheless, E_2_BSA decreased the magnitude of the preovulatory surge of LH induced by E_2_, not only delaying the onset of the surge of LH, but also resulting in an early termination of the preovulatory-like surge of LH, and to some extent reducing the amount of LH released. It is not clear why the decrease in the amount of LH released by E_2_BSA was more apparent in ewes highly responsive to E_2 _than in ewes with a lower responsiveness to E_2_. If as proposed, E_2_BSA is reducing responsiveness of the pituitary gland to GnRH, it is more likely that E_2_BSA could override the GnRH input during the beginning and the end of the surge of LH, when secretion of GnRH is not at the maximum concentration [1,4,8,16,17,19]. Notwithstanding the expected increase in the number of GnRH receptors, the net effect of E_2_BSA was a reduction in the magnitude of the preovulatory-like surge of LH. This implies a predominant role of the membrane-initiated actions of E_2 _in the mediation of the negative feedback effect on LH secretion compared with a minor contribution, by itself, on the synthesis of GnRH receptor. This interpretation is consistent with the slight increase in the number of GnRH receptors induced by E_2_BSA, compared with that induced by E_2_, and agrees with other experimental paradigms where equimolar doses of conjugated or un-conjugated E_2 _induced a similar decrease in LH secretion in ewes, [30] and in primary cultures of ovine pituitary cells [13]; whereas in the same cells, higher doses of E_2_BSA were required to increase the number of GnRH receptors [39].

Although regulation of synthesis and/or secretion of GnRH by E_2_BSA were not evaluated during the expected time of the preovulatory-like surge of LH, we do not think that the effects were mediated via the hypothalamus. In support of this notion: a) E_2_BSA did not modulate the pulsatile profile of GnRH (at least for the first 4 h after administration), b) conjugated E_2 _was stable in blood (at least for 24 h), and c) administration of a high dose of E_2_BSA just before the preovulatory-like surge of LH induced by E_2 _decreased the amount of LH released. Therefore we suggest that the actions of E_2_BSA on LH secretion are the result of a direct action on the pituitary gland.

## Conclusions

The rapid suppression of LH secretion induced by E_2_BSA is mediated only via a direct action on the pituitary gland. E_2 _acting via a membrane-initiated pathway contributes to the E_2_-induced increase in the number of GnRH receptors. Administration of E_2_BSA near the beginning of the pre-ovulatory LH surge delays and reduces the magnitude of the surge.

## Competing interests

The authors declare that they have no competing interests.

## Authors' contributions

All authors contributed to the design of the study and revised critically the manuscript. RLA acquisition of data for experiments 2 and 3. AEO, and FJK acquisition of data for experiment 1 and data interpretation, ERW acquisition of data for experiment 1, data interpretation, and radioimmuno assays for GnRH. TMN acquisition of data for experiments 2 and 3 and data interpretation. JAAA acquisition of data for experiments 1, 2, and 3, data interpretation, radioimmuno assays for LH and E_2_, quantification of GnRH receptors, data analysis, and drafted the manuscript. All authors read and approved the manuscript.

## References

[B1] Clarke IJ Variable patterns of gonadotropin-releasing hormone secretion during the estrogen-induced luteinizing hormone surge in ovariectomized ewes Endocrinology 1993 133 1624 1632 10.1210/en.133.4.1624 8404603

[B2] Nett TM Crowder ME Wise ME Role of estradiol in inducing an ovulatory-like surge of luteinizing hormone in sheep Biol Reprod 1984 30 1208 1215 10.1095/biolreprod30.5.1208 6329340

[B3] Evans NP Dahl GE Mauger DT Padmanabhan V Thrun LA Karsch FJ Does estradiol induce the preovulatory gonadotropin-releasing hormone (GnRH) surge in the ewe by inducing a progressive change in the mode of operation of the GnRH neurosecretory system Endocrinology 1995 136 5511 5519 10.1210/en.136.12.5511 7588302

[B4] Evans NP Dahl GE Padmanabhan V Thrun LA Karsch FJ Estradiol requirements for induction and maintenance of the gonadotropin-releasing hormone surge: implications for neuroendocrine processing of the estradiol signal Endocrinology 1997 138 5408 5414 10.1210/en.138.12.5408 9389526

[B5] Evans NP Dahl GE Glover BH Karsch FJ Central regulation of pulsatile gonadotropin-releasing hormone (GnRH) secretion by estradiol during the period leading up to the preovulatory GnRH surge in the ewe Endocrinology 1994 134 1806 1811 10.1210/en.134.4.1806 8137746

[B6] Clarke IJ Cummins JT Crowder ME Nett TM Pituitary receptors for gonadotropin-releasing hormone in relation to changes in pituitary and plasma gonadotropins in ovariectomized hypothalamo/pituitary-disconnected ewes. II. A marked rise in receptor number during the acute feedback effects of estradiol Biol Reprod 1988 39 349 354 10.1095/biolreprod39.2.349 2846084

[B7] Gregg DW Allen MC Nett TM Estradiol-induced increase in number of gonadotropin-releasing hormone receptors in cultured ovine pituitary cells Biol Reprod 1990 43 1032 1036 10.1095/biolreprod43.6.1032 1963321

[B8] Moenter SM Caraty A Karsch FJ The estradiol-induced surge of gonadotropin-releasing hormone in the ewe Endocrinology 1990 127 1375 1384 10.1210/endo-127-3-1375 2201536

[B9] Herbison AE Neurochemical identity of neurones expressing oestrogen and androgen receptors in sheep hypothalamus J Reprod Fertil Suppl 1995 49 271 283 7623319

[B10] Scott CJ Tilbrook AJ Rawson JA Clarke IJ Gonadal steroid receptors in the regulation of GnRH secretion in farm animals Anim Reprod Sci 2000 60-61 313 326 10.1016/S0378-4320(00)00103-2 10844203

[B11] Clarke IJ Cummins JT Direct pituitary effects of estrogen and progesterone on gonadotropin secretion in the ovariectomized ewe Neuroendocrinology 1984 39 267 274 10.1159/000123990 6438545

[B12] Gregg DW Nett TM Direct effects of estradiol-17 beta on the number of gonadotropin-releasing hormone receptors in the ovine pituitary Biol Reprod 1989 40 288 293 10.1095/biolreprod40.2.288 2541815

[B13] Arreguin-Arevalo JA Nett TM A nongenomic action of 17beta-estradiol as the mechanism underlying the acute suppression of secretion of luteinizing hormone Biol Reprod 2005 73 115 122 10.1095/biolreprod.105.040329 15772257

[B14] Mercer JE Phillips DJ Clarke IJ Short-term regulation of gonadotropin subunit mRNA levels by estrogen: studies in the hypothalamo-pituitary intact and hypothalamo-pituitary disconnected ewe J Neuroendocrinol 1993 5 591 596 10.1111/j.1365-2826.1993.tb00526.x 8680429

[B15] Clarke IJ Pompolo S Scott CJ Rawson JA Caddy D Jakubowska AE Pereira AM Cells of the arcuate nucleus and ventromedial nucleus of the ovariectomized ewe that respond to oestrogen: a study using Fos immunohistochemistry J Neuroendocrinol 2001 13 934 941 10.1046/j.1365-2826.2001.00694.x 11737551

[B16] Moenter SM Caraty A Locatelli A Karsch FJ Pattern of gonadotropin-releasing hormone (GnRH) secretion leading up to ovulation in the ewe: existence of a preovulatory GnRH surge Endocrinology 1991 129 1175 1182 10.1210/endo-129-3-1175 1874164

[B17] Caraty A Fabre-Nys C Delaleu B Locatelli A Bruneau G Karsch FJ Herbison A Evidence that the mediobasal hypothalamus is the primary site of action of estradiol in inducing the preovulatory gonadotropin releasing hormone surge in the ewe Endocrinology 1998 139 1752 1760 10.1210/en.139.4.1752 9528959

[B18] Clarke IJ Evidence that the switch from negative to positive feedback at the level of the pituitary gland is an important timing event for the onset of the preovulatory surge in LH in the ewe J Endocrinol 1995 145 271 282 10.1677/joe.0.1450271 7616160

[B19] Clarke IJ Multifarious effects of estrogen on the pituitary gonadotrope with special emphasis on studies in the ovine species Arch Physiol Biochem 2002 110 62 73 10.1076/apab.110.1.62.898 11935402

[B20] Katzenellenbogen BS Katzenellenbogen JA Estrogen receptor transcription and transactivation: Estrogen receptor alpha and estrogen receptor beta: regulation by selective estrogen receptor modulators and importance in breast cancer Breast Cancer Res 2000 2 335 344 10.1186/bcr78 11250726 PMC138655

[B21] Hall JM Couse JF Korach KS The multifaceted mechanisms of estradiol and estrogen receptor signaling J Biol Chem 2001 276 36869 36872 10.1074/jbc.R100029200 11459850

[B22] Levin ER Cell localization, physiology, and nongenomic actions of estrogen receptors J Appl Physiol 2001 91 1860 1867 11568173 10.1152/jappl.2001.91.4.1860

[B23] Bjornstrom L Sjoberg M Mechanisms of estrogen receptor signaling: convergence of genomic and nongenomic actions on target genes Mol Endocrinol 2005 19 833 842 10.1210/me.2004-0486 15695368

[B24] Razandi M Pedram A Greene GL Levin ER Cell membrane and nuclear estrogen receptors (ERs) originate from a single transcript: studies of ERalpha and ERbeta expressed in Chinese hamster ovary cells Mol Endocrinol 1999 13 307 319 10.1210/me.13.2.307 9973260

[B25] Razandi M Pedram A Levin ER Estrogen signals to the preservation of endothelial cell form and function J Biol Chem 2000 275 38540 38546 10.1074/jbc.M007555200 10988297

[B26] Wade CB Robinson S Shapiro RA Dorsa DM Estrogen receptor (ER)alpha and ERbeta exhibit unique pharmacologic properties when coupled to activation of the mitogen-activated protein kinase pathway Endocrinology 2001 142 2336 2342 10.1210/en.142.6.2336 11356680

[B27] Dos Santos EG Dieudonne MN Pecquery R Le Moal V Giudicelli Y Lacasa D Rapid nongenomic E2 effects on p42/p44 MAPK, activator protein-1, and cAMP response element binding protein in rat white adipocytes Endocrinology 2002 143 930 940 10.1210/en.143.3.930 11861515

[B28] Chen DB Bird IM Zheng J Magness RR Membrane estrogen receptor-dependent extracellular signal-regulated kinase pathway mediates acute activation of endothelial nitric oxide synthase by estrogen in uterine artery endothelial cells Endocrinology 2004 145 113 125 10.1210/en.2003-0547 14512434

[B29] Wong JK Le HH Zsarnovszky A Belcher SM Estrogens and ICI182,780 (Faslodex) modulate mitosis and cell death in immature cerebellar neurons via rapid activation of p44/p42 mitogen-activated protein kinase J Neurosci 2003 23 4984 4995 12832521 10.1523/JNEUROSCI.23-12-04984.2003 PMC6741198

[B30] Arreguin-Arevalo JA Nett TM A nongenomic action of estradiol as the mechanism underlying the acute suppression of secretion of luteinizing hormone in ovariectomized ewes Biol Reprod 2006 74 202 208 10.1095/biolreprod.105.044685 16207838

[B31] Korenman SG Stevens RH Carpenter LA Robb M Niswender GD Sherman BM Estradiol radioimmunoassay without chromatography: procedure, validation and normal values J Clin Endocrinol Metab 1974 38 718 720 10.1210/jcem-38-4-718 4820671

[B32] Nett TM Crowder ME Moss GE Duello TM GnRH-receptor interaction. V. Down-regulation of pituitary receptors for GnRH in ovariectomized ewes by infusion of homologous hormone Biol Reprod 1981 24 1145 1155 6268204

[B33] Wagner TO Adams TE Nett TM GNRH interaction with anterior pituitary. I. Determination of the affinity and number of receptors for GNRH in ovine anterior pituitary Biol Reprod 1979 20 140 149 10.1095/biolreprod20.2.140 222362

[B34] Caraty A Locatelli A Moenter S Karsch F Sampling of hypophyseal portal blood of the conscious sheep for direct monitoring of hypothalamic neurosecretory substances Methods in Neuroscience 1994 20 162 183

[B35] Caraty A Locatelli A Schanbacher B [Augmentation, by naloxone, of the frequency and amplitude of LH-RH pulses in hypothalamo-hypophyseal portal blood in the castrated ram] C R Acad Sci III 1987 305 369 374 3113690

[B36] Niswender GD Reichert LE Jr Midgley AR Jr Nalbandov AV Radioimmunoassay for bovine and ovine luteinizing hormone Endocrinology 1969 84 1166 1173 10.1210/endo-84-5-1166 5813408

[B37] Moenter SM Brand RM Midgley AR Karsch FJ Dynamics of gonadotropin-releasing hormone release during a pulse Endocrinology 1992 130 503 510 10.1210/en.130.1.503 1727719

[B38] Arreguin-Arevalo JA Davis TL Nett TM Differential modulation of gonadotropin secretion by selective estrogen receptor 1 and estrogen receptor 2 agonists in ovariectomized ewes Biol Reprod 2007 77 320 328 10.1095/biolreprod.107.060046 17429013

[B39] Davis TL Clay CM Nett TM Membrane impermeable estradiol-induced increase in number of gonadotropin-releasing hormone receptor in cultured ovine pituitary cells39th Annual Meeting Society for the Study of Reproduction 2006 95 96

[B40] SAS SAS User's Guide SC 1987 NC: Statistical Analysis System Institute, Inc.

[B41] Iqbal J Latchoumanin O Sari IP Lang RJ Coleman HA Parkington HC Clarke IJ Estradiol-17beta inhibits gonadotropin-releasing hormone-induced Ca2+ in gonadotropes to regulate negative feedback on luteinizing hormone release Endocrinology 2009 150 4213 4220 10.1210/en.2009-0092 19477939

[B42] Iqbal J Latchoumanin O Clarke IJ Rapid in vivo effects of estradiol-17beta in ovine pituitary gonadotropes are displayed by phosphorylation of extracellularly regulated kinase, serine/threonine kinase, and 3',5'-cyclic adenosine 5'-monophosphate-responsive element-binding protein Endocrinology 2007 148 5794 5802 10.1210/en.2007-0986 17823264

[B43] Caraty A Locatelli A Martin GB Biphasic response in the secretion of gonadotrophin-releasing hormone in ovariectomized ewes injected with oestradiol J Endocrinol 1989 123 375 382 10.1677/joe.0.1230375 2691622

[B44] Evans NP Dahl GE Mauger D Karsch FJ Estradiol induces both qualitative and quantitative changes in the pattern of gonadotropin-releasing hormone secretion during the presurge period in the ewe Endocrinology 1995 136 1603 1609 10.1210/en.136.4.1603 7895670

[B45] Clarke IJ Cummins JT Crowder ME Nett TM Long-term negative feedback effects of oestrogen and progesterone on the pituitary gland of the long-term ovariectomized ewe J Endocrinol 1989 120 207 214 10.1677/joe.0.1200207 2538532

[B46] Crowder ME Nett TM Pituitary content of gonadotropins and receptors for gonadotropin-releasing hormone (GnRH) and hypothalamic content of GnRH during the periovulatory period of the ewe Endocrinology 1984 114 234 239 10.1210/endo-114-1-234 6317346

[B47] Albarracin CT Kaiser UB Chin WW Isolation and characterization of the 5'-flanking region of the mouse gonadotropin-releasing hormone receptor gene Endocrinology 1994 135 2300 2306 10.1210/en.135.6.2300 7988412

[B48] Campion CE Turzillo AM Clay CM The gene encoding the ovine gonadotropin-releasing hormone (GnRH) receptor: cloning and initial characterization Gene 1996 170 277 280 10.1016/0378-1119(96)00042-X 8666259

[B49] Fan NC Peng C Krisinger J Leung PC The human gonadotropin-releasing hormone receptor gene: complete structure including multiple promoters, transcription initiation sites, and polyadenylation signals Mol Cell Endocrinol 1995 107 R1 8 10.1016/0303-7207(94)03460-B 7768323

[B50] Kakar SS Musgrove LC Devor DC Sellers JC Neill JD Cloning, sequencing, and expression of human gonadotropin releasing hormone (GnRH) receptor Biochem Biophys Res Commun 1992 189 289 295 10.1016/0006-291X(92)91556-6 1333190

